# Association between unintentional firearm injury characteristics and deaths in adolescents

**DOI:** 10.1186/s40621-024-00543-z

**Published:** 2024-11-04

**Authors:** Ashley A. Hollo, Mairead Dillon, Jennifer A. Hoffmann, Ashley Blanchard, Maya Haasz

**Affiliations:** 1 Department of Pediatrics, University of Colorado School of Medicine, Aurora, CO USA; 2 Division of Emergency Medicine, Ann & Robert H. Lurie Children’s Hospital of Chicago, Chicago, IL USA; 3 Northwestern University Feinberg School of Medicine, Chicago, IL USA; 4 Department of Emergency Medicine, Columbia University Vagelos College of Physicians and Surgeons, New York, NY USA; 5 Department of Pediatrics, Section of Emergency Medicine, Children’s Hospital Colorado, University of Colorado School of Medicine, Aurora, CO USA

**Keywords:** Gun violence, Public health, Injury prevention, Pediatrics, Unintentional injury

## Abstract

**Background:**

Approximately 15% of pediatric firearm injuries are unintentional. While demographic characteristics of unintentional firearm injuries have been described, the relationship between injury characteristics and mortality is not well understood. In this study, we identified injury characteristics associated with fatality among unintentional firearm injuries in adolescents.

**Methods:**

We conducted a retrospective (May 2022-May 2023) cross-sectional study of unintentional firearm injuries among adolescents 12–17 years old using the Gun Violence Archive. Variables included victim age and sex, shooter age and sex, injury location, injury circumstance, number of firearms, type of firearm, firearm owner, census region, and shooter relationship to the victim. Logistic regression was used to identify variables associated with fatality.

**Results:**

Of 319 unintentional incidents, 212 (66.5%) were non-fatal and 107 (34.5%) were fatal. Of all shootings, 176 (55.2%) occurred in a residence. A shooter was identified in 256 (80.3%) cases; 43.0% of these were a peer of the victim. The adjusted odds of fatality were higher when a peer was the shooter (aOR 5.38, 95% CI 2.57, 11.80) compared to self-inflicted injury and when the shooting took place in the victim’s residence (aOR 2.87, 95% CI 1.07, 7.88) or another residence (aOR 3.03, 95% CI 1.45, 6.67) versus a public location (Fig. 1).

**Conclusions:**

Unintentional firearm injuries were more likely fatal when a peer was the shooter and when the shooting occurred at a residence. This amplifies the importance of safe home firearm storage and exploring other evidence-based approaches to decreasing youth access to firearms.

## Introduction

Approximately 15% of pediatric firearm injuries (fatal and non-fatal) are unintentional Naik-Mathuria et al. (2023). Previous cross-sectional studies of pediatric unintentional firearm fatalities using National Violent Death Reporting System (NVDRS) data have found that victims are predominantly male, 66–85% of incidents occurred in a home, and more than half of injuries were inflicted by others (Wilson et al. 2023; Vaishnav et al. 2023; Hemenway at al. 2010; Hemenway and Solnick 2015). More detailed data on injury characteristics and non-fatal injuries in adolescents, and the relationship between specific characteristics of unintentional firearm injuries and risk of fatality, is lacking. In this study, we examined injury characteristics associated with fatality among unintentional firearm injuries in adolescents 12–17 years old.

## Methods

We conducted a retrospective (May 2022-May 2023) cross-sectional study of unintentional firearm injuries among adolescents 12–17 years old using data from the Gun Violence Archive (GVA) (Gun Violence Archive 2024). The GVA is a publicly available national database of firearm-related incidents collected from law enforcement, media, government, and commercial sources that includes associated media and police reports (Gun Violence Archive 2024). It provides granular data regarding incident location, collects data near real-time allowing for more timely and relevant analyses, and has high correlation (> 0.95) with CDC data for interpersonal violence (Johnson et al. 2021). This study was deemed exempt by the University of Colorado Institutional Review Board.

One investigator (AH) reviewed all records; approximately 10% of records were reviewed by a second investigator (MH), with discrepancies resolved by consensus. Logistic regression was used to examine injury characteristics associated with fatality. Initial variables included victim age and sex, shooter age and sex, injury location, injury circumstance, number of firearms, type of firearm, firearm owner, census region, and shooter relationship to the victim. Missing data were categorized as “unspecified” and included in models. Backward stepwise selection was used to identify a model that minimized Akaike Information Criterion. The final model included victim sex, shooter sex, injury location, number of firearms, type of firearm, and shooter relationship to the victim. Firth’s penalized logistic regression was used to mitigate the issues of complete separation and small sample size. Analysis was performed using R version 4.3.1 (R Core Team 2016).

## Results

Of 319 unintentional shooting incidents, 212 (66.5%) were non-fatal and 107 (34.5%) were fatal. The median victim age was 15.0 years old (IQR 14.0, 16.0); 71.8% of victims were male and the injury was self-inflicted in 21.3% of incidents (Table 1). More than half of shootings occurred in a place of residence. In 80.3% of cases, a shooter was identified. Of known shooters (80.3%), 43.0% were a peer of the injured adolescent.

**Table 1:** Characteristics of fatal and nonfatal unintentional firearm injuries in adolescents, May 2022 to May 2023

	Overall (*N* = 319)	Fatal (*N* = 107)	Non-fatal (*N* = 212)
**Victim age**			
12	33 (10.3%)	11 (10.3%)	22 (10.4%)
13	32 (10.0%)	8 (7.5%)	24 (11.3%)
14	39 (12.2%)	17 (15.9%)	22 (10.4%)
15	58 (18.2%)	24 (22.4%)	34 (16.0%)
16	52 (16.3%)	16 (15.0%)	36 (17.0%)
17	60 (18.8%)	22 (20.6%)	38 (17.9%)
Unspecified teen	45 (14.1%)	9 (8.4%)	36 (17.0%)
**Victim sex**			
Male	229 (71.8%)	83 (77.6%)	146 (68.9%)
Female	68 (21.3%)	24 (22.4%)	44 (20.8%)
Unspecified	22 (6.9%)	0 (0%)	22 (10.4%)
**Shooter age**			
0–11	5 (1.6%)	1 (0.9%)	4 (1.9%)
12–18	198 (62.1%)	77 (72.0%)	121 (57.1%)
Adult, 19 or older	43 (13.5%)	18 (16.8%)	25 (11.8%)
Unspecified	73 (22.9%)	11 (10.3%)	62 (29.2%)
**Shooter sex**			
Male	214 (67.1%)	82 (76.6%)	132 (62.3%)
Female	14 (4.4%)	10 (9.3%)	4 (1.9%)
Both male and female or unspecified	91 (28.5%)	15 (14.0%)	76 (35.8%)
**Location**			
Victim’s home	37 (11.6%)	13 (12.1%)	24 (11.3%)
Other home	139 (43.6%)	61 (57.0%)	78 (36.8%)
Public location	66 (20.7%)	12 (11.2%)	54 (25.5%)
Other/unspecified location	77 (24.1%)	21 (19.6%)	56 (26.4%)
**Circumstance**			
Cleaning gun or other accidental	271 (85.0%)	98 (91.6%)	173 (81.6%)
Hunting/sport shooting	11 (3.4%)	4 (3.7%)	7 (3.3%)
Stray bullet	37 (11.6%)	5 (4.7%)	32 (15.1%)
**Number of firearms**			
1	299 (93.7%)	96 (89.7%)	203 (95.8%)
2 or more	20 (6.3%)	11 (10.3%)	9 (4.2%)
**Type of firearm involved**			
Handgun	76 (23.8%)	27 (25.2%)	49 (23.1%)
Long gun	17 (5.3%)	6 (5.6%)	11 (5.2%)
Unspecified	226 (70.8%)	74 (69.2%)	152 (71.7%)
**Firearm owner**			
Self (shooter)	61 (19.1%)	24 (22.4%)	37 (17.5%)
Family member	35 (11.0%)	15 (14.0%)	20 (9.4%)
Peer	12 (3.8%)	8 (7.5%)	4 (1.9%)
Other/unspecified	211 (66.1%)	60 (56.1%)	151 (71.2%)
**Census region**			
Midwest	79 (24.8%)	20 (18.7%)	59 (27.8%)
Northeast	40 (12.5%)	12 (11.2%)	28 (13.2%)
South	158 (49.5%)	59 (55.1%)	99 (46.7%)
West	42 (13.2%)	16 (15.0%)	26 (12.3%)
**Shooter’s relationship to victim**			
Self as shooter	68 (21.3%)	15 (14.0%)	53 (25.0%)
Family member as shooter	35 (11.0%)	13 (12.1%)	22 (10.4%)
Peer as shooter	110 (34.5%)	59 (55.1%)	51 (24.1%)
Other shooter	106 (33.2%)	20 (18.7%)	86 (40.6%)

**Fig. 1:**
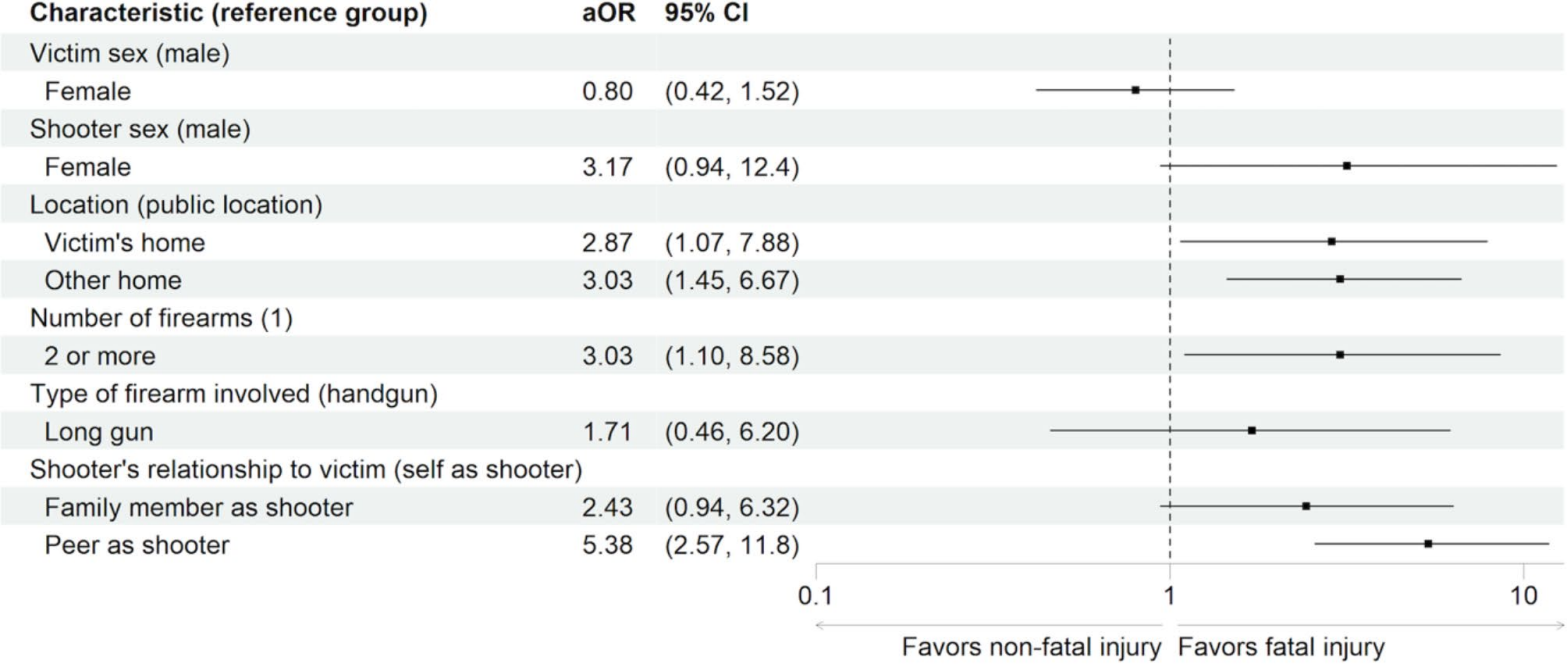
Association of Injury Characteristics and Fatality: Adjusted odds ratio (aOR) of fatality for each characteristic with associated 95% confidence intervals. The reference group appears in parentheses. The vertical line in the forest plot denotes OR=1

The adjusted odds of fatality were higher when a peer was the shooter (aOR 5.38, 95% CI 2.57, 11.80) compared to self-inflicted injury, and when more than one firearm was present (aOR 3.03, 95% CI 1.10, 8.60) compared with one firearm. The adjusted odds were also higher for shootings in the victim’s home (aOR 2.87, 95% CI 1.07, 7.88) or in another home (aOR 3.03, 95% CI 1.45, 6.67) versus a public location (Fig. 1).

## Discussion

In this cross-sectional study of unintentional injuries among adolescents 12–17 years old, more than two-thirds of victims were male and over half of injuries occurred in a home, complimenting NVDRS data and filling a crucial knowledge gap around non-fatal injuries (Wilson et al. 2023; Vaishnav et al. 2023). Unlike NVDRS data, which looked only at fatal injuries and showed that approximately half were self-inflicted, one in five injuries (both fatal and non-fatal) in this study were self-inflicted (Wilson et al. 2023; Vaishnav et al. 2023; Hemenway at al. 2010; Hemenway and Solnick 2015). This discrepancy may be partly due to the inclusion of younger age groups (ages 0–5), which have higher rates of self-inflicted injuries, in NVDRS data (Wilson et al. 2023; Vaishnav et al. 2023).

Our finding that unintentional firearm injuries are more likely to be fatal when they occur in a place of residence is particularly concerning. Approximately 40% of US households with children have a firearm, and 4.6 million US children live in homes with firearms stored in the least safe way (unlocked and loaded), increasing the risk of unintentional injury, suicide, and homicide (Azrael et al. 2018; Miller and Azrael 2022). In a survey of adolescents, 44.5% reported access to a firearm, and 68% of these adolescents reported home firearm access (Haasz et al. 2023). Our work amplifies the importance of decreasing youth access to firearms among firearm-owning households through various methods including safe storage. Child Access Prevention laws, state-level laws that impose a penalty for improperly stored household firearms, are one example of an evidence-based approach to reducing pediatric unintentional firearm injuries (Azad et al. 2020; RAND Corporation 2024). Further prospective work is needed to determine whether decreasing access to home firearms decreases unintentional firearm injuries in general, and fatal injuries in particular (Wilson et al. 2023; R Core Team 2016).

We also found that fatal injury is associated with multiple firearms and peers as shooters. Work to date has primarily focused on parental education around firearm safety (Haasz et al. 2024; Burch et al. 2023; Fraser Doh et al. 2024). Future work should explore whether educating adolescents about firearm safety can decrease the incidence and fatality of unintentional firearm injuries (Haasz et al. 2022).

These findings should be considered in the context of several limitations. First, the GVA is not a government-regulated data source and not all pediatric firearm injuries are captured, although correlation with Centers for Disease Control and Prevention epidemiologic data is high (Johnson et al. 2021). Second, some incident characteristics were missing. This lack of detail limits their helpfulness in formulating prevention strategies. Finally, there is potential for miscategorization of intent and other injury characteristics, as the GVA is a secondary data source. Despite these limitations, this study provides an important understanding of specific characteristics associated with fatal unintentional firearm injuries in adolescents that may help support policies and research to prevent firearm deaths.

## Data Availability

The datasets analyzed during the current study are available in the Gun Violence Archive repository, https://www.gunviolencearchive.org/. All data generated during this study are included in this published article.

## Abbreviations

Gun Violence Archive (GVA): Publicly available national database of firearm-related incidents
National Violent Death Reporting System (NVDRS): Surveillance system that documents violent injuries and is maintained by the Centers for Disease Control and Prevention

## References

[CR1] Azad HA, Monuteaux MC, Rees CA, Siegel M, Mannix R, Lee LK, et al. Child Access Prevention Firearm laws and Firearm fatalities among children aged 0 to 14 years, 1991–2016. JAMA Pediatr. 2020;174(5):1–8. 32119063 10.1001/jamapediatrics.2019.6227 PMC7052788

[CR2] Azrael D, Cohen J, Salhi C, Miller M. Firearm Storage in Gun-owning households with children: results of a 2015 National Survey. J Urban Health Bull N Y Acad Med. 2018;95(3):295–304. 10.1007/s11524-018-0261-7 PMC5993703 29748766

[CR3] Burch C, Webb A, Jorge E, King B, Nichols M, Monroe K. Safe at home: prevention of pediatric unintentional injuries. Inj Epidemiol. 2023;10(Suppl 1):30. 37400908 10.1186/s40621-023-00442-9 PMC10318633

[CR5] Fraser Doh K, Bishop Z, Gillings T, Johnson J, Boy A, Waris RS, et al. Receptivity of providing firearm safety storage devices to parents along with firearms safety education. Front Public Health. 2024;12:1352400. 38577291 10.3389/fpubh.2024.1352400 PMC10991684

[CR6] Gun Violence Archive [Internet]. [cited 2024 Feb 22]. https://www.gunviolencearchive.org/

[CR7] Haasz M, Boggs JM, Beidas RS, Betz ME, Firearms. Physicians, families, and kids: finding words that work. J Pediatr. 2022;247:133–7. 35605644 10.1016/j.jpeds.2022.05.029

[CR8] Haasz M, Myers MG, Rowhani-Rahbar A, Zimmerman MA, Seewald L, Sokol RL, et al. Firearms availability among high-school Age Youth with recent depression or suicidality. Pediatrics. 2023;151(6):e2022059532. 37212021 10.1542/peds.2022-059532 PMC10233739

[CR9] Haasz M, Betz ME, Ambroggio L, Cafferty R, King CA, Wong S et al. Acceptability and feasibility of video-based firearm safety education in a Colorado emergency department for caregivers of adolescents in firearm-owning households. Inj Prev J Int Soc Child Adolesc Inj Prev. 2024;ip–2023. 10.1136/ip-2023-045204 PMC11757795 39053924

[CR10] Hemenway D, Solnick SJ. Children and unintentional firearm death. Inj Epidemiol. 2015;2(1):26. 26478854 10.1186/s40621-015-0057-0 PMC4602049

[CR11] Hemenway D, Barber C, Miller M. Unintentional firearm deaths: a comparison of other-inflicted and self-inflicted shootings. Accid Anal Prev. 2010;42(4):1184–8. 20441829 10.1016/j.aap.2010.01.008

[CR12] Johnson BT, Sisti A, Bernstein M, Chen K, Hennessy EA, Acabchuk RL, et al. Community-level factors and incidence of gun violence in the United States, 2014–2017. Soc Sci Med 1982. 2021;280:113969. 10.1016/j.socscimed.2021.113969 34111630

[CR13] Miller M, Azrael D. Firearm storage in US Households with children: findings from the 2021 National Firearm Survey. JAMA Netw Open. 2022;5(2):e2148823. 35191973 10.1001/jamanetworkopen.2021.48823 PMC8864510

[CR14] Naik-Mathuria BJ, Cain CM, Alore EA, Chen L, Pompeii LA. Defining the full spectrum of Pediatric Firearm Injury and Death in the United States: it is even worse than we think. Ann Surg. 2023;278(1):10. 36825500 10.1097/SLA.0000000000005833 PMC10249597

[CR15] R Core Team. R: A Language and Environment for Statistical Computing. [Internet]. Vienna, Austria. 2016. https://www.R-project.org/

[CR4] RAND Corporation. Effects of Child-Access Prevention Laws on Unintentional. Injuries and Deaths [Internet]. [cited 2024 Sep 30]. https://www.rand.org/research/gun-policy/analysis/child-access-prevention/unintentional-injuries.html

[CR16] Vaishnav A, Smith GA, Badeti J, Michaels NL. An epidemiological study of unintentional pediatric firearm fatalities in the USA, 2009–2018. Inj Epidemiol. 2023;10(1):25. 37357309 10.1186/s40621-023-00438-5 PMC10291813

[CR17] Wilson RF, Mintz S, Blair JM, Betz CJ, Collier A, Fowler KA. Unintentional Firearm Injury deaths among children and adolescents aged 0–17 years - National Violent Death Reporting System, United States, 2003–2021. MMWR Morb Mortal Wkly Rep. 2023;72(50):1338–45. 38096119 10.15585/mmwr.mm7250a1 PMC10727142

